# Gut Bacterial Composition and Nutritional Implications in Mexican and Spanish Individuals with Inflammatory Bowel Disease Compared to Healthy Controls

**DOI:** 10.3390/ijms252211887

**Published:** 2024-11-05

**Authors:** Ricardo García-Gamboa, Osiris Díaz-Torres, Misael Sebastián Gradilla-Hernández, Vicente Pérez-Brocal, Andrés Moya, Marisela González-Avila

**Affiliations:** 1 Tecnologico de Monterrey, Escuela de Medicina y Ciencias de la Salud, Av. General Ramon Corona 2514, Nuevo Mexico, Zapopan 45138, Jalisco, Mexico; 2 Tecnologico de Monterrey, Escuela de Ingenieria y Ciencias, Laboratorio de Sostenibilidad y Cambio Climático, Av. General Ramon Corona 2514, Zapopan 45138, Jalisco, Mexico; 3 Fundación para el Fomento de la Investigación Sanitaria y Biomédica de la Comunitat Valenciana (FISABIO), Av. de Cataluña 21, 46020 València, Spain; 4 Consorcio de Investigación Biomédica en Red de Epidemiología y Salud Pública (CIBERSEP), c/Monforte de Lemos 3-5, Pabellón 11, 28029 Madrid, Spain; 5 Integrative Systems Biology Institute (I2SysBio), Universitat de València and Consejo Superior de Investigaciones Científicas (CSIC), c/Catedrático José Beltrán 2, 46980 Paterna, València, Spain; 6 Centro de Investigación y Asistencia en Tecnología y Diseño del Estado de Jalisco A.C., Av. Normalistas No. 800, col Colinas de la Normal, Guadalajara 44270, Jalisco, Mexico

**Keywords:** inflammatory bowel disease, gut microbiota, Bacillota/Bacteroidota (Firmicutes/Bacteroidetes), nutritional influence, 16S rRNA gene sequencing

## Abstract

The intestinal microbiota plays a key role in the pathogenesis of inflammatory bowel disease (IBD), with its composition varying based on geographic location and dietary factors. This study was performed to examine and compare the bacterial composition of the gut microbiota in Mexican and Spanish individuals with IBD and healthy controls, while also considering the nutritional aspects. This study involved 79 individuals with IBD and healthy controls from Mexico and Spain. The fecal microbiota composition was analyzed using 16S rRNA gene sequencing, and the dietary intake and anthropometric measurements were collected. Alpha diversity analysis revealed a lower Chao1 index of the bacterial genera in the IBD groups. Beta diversity analysis showed significant differences in the bacterial composition, suggesting inter-individual variability within the healthy and IBD groups. Additionally, the relative abundance of the bacterial genera varied across the four groups. *Faecalibacterium* was more abundant in the IBD groups; *Prevotella* was found exclusively in the Mexican groups, and *Akkermansia* was found only in the Spanish groups. *Akkermansia* was positively correlated with meat and protein intake, *Prevotella* with lean mass, and *Bacteroides* with calorie intake. These findings highlight the importance of considering geographic and nutritional factors in future research on the gut microbiome’s role in IBD pathogenesis.

## 1. Introduction

Inflammatory bowel disease (IBD), which includes Crohn’s disease (CD) and ulcerative colitis (UC), is a chronic gastrointestinal disorder characterized by recurrent episodes of diarrhea, hematochezia, fever, abdominal pain, and other clinical manifestations [1]. CD can affect any part of the gastrointestinal tract from the mouth to the anus and is marked by transmural inflammation, potentially leading to complications such as strictures and fistulas. By contrast, UC primarily involves the continuous inflammation of the colonic mucosa and submucosa, with CD presenting discontinuous lesions and UC exhibiting continuous areas of inflammation [2]. The etiology of IBD is complex and multifactorial, involving genetic predispositions, immune system dysregulation, environmental influences, and gut microbiota interactions [3]. Notably, the global incidence of IBD has risen significantly over the past 50 years, particularly in industrialized nations adopting Western lifestyles [4].

Epidemiological data suggest a significant geographic influence on the etiology of IBD, with a higher incidence in industrialized countries, particularly those adopting Western lifestyles [5]. In Mexico, the incidence is estimated at 0.16 cases per 100,000 person-years for UC and 0.04 cases per 100,000 person-years for CD [6]. By contrast, Spain reports higher incidence rates, with 9.1 cases per 100,000 person-years for UC and 7.5 cases per 100,000 person-years for CD [7]. Socioeconomic factors, urbanization, and dietary patterns associated with Westernization have also been linked to the rising incidence of IBD in various populations [3].

IBD is closely associated with the human gut microbiome—the microbial communities within the gastrointestinal tract—which plays a crucial role in human physiology and disease [8]. The intestines harbor a diverse microbiota, including bacterial, fungal, and other microbial communities [9]. Individuals with IBD often exhibit intestinal dysbiosis, characterized by reduced microbial diversity and an altered community structure [10]. For example, a lower abundance of Bacillota and Actinomycetota is noted in both patients with CD and those with UC [11], while specific genera such as *Roseburia* and *Faecalibacterium* are significantly reduced in CD [12]. By contrast, *Ruminococcus gnavus* is increased in these patients [13]. Understanding the composition of the gut microbiota is essential for understanding human health and disease, including IBD. Moreover, the interaction between dietary components, such as fiber and protein, and the gut microbiome can significantly affect inflammatory processes in IBD, highlighting the importance of diet in disease management [14].

The intestinal microbiota is involved in crucial physiological processes such as digestion and metabolism, optimizing energy extraction from dietary components. Additionally, bacteria within the intestinal microbiota play a significant role in regulating intestinal inflammation, a key factor that is disrupted in IBD [15]. Diet plays a pivotal role in shaping the gut microbiota, and the nutritional status is often compromised in individuals with IBD. Well-documented dietary interventions can significantly improve the quality of life for these individuals. Many patients require an increased caloric intake because of malnutrition, a common complication associated with a suboptimal body composition [14]. Certain dietary patterns, such as the Mediterranean diet, have been linked to better outcomes in patients with IBD, suggesting that dietary modifications may serve as an adjunct therapy. Anthropometric parameters, such as weight and height, are considered reliable indicators of the nutritional status in individuals with IBD [16]. High-throughput sequencing has revolutionized the analysis of the taxonomic composition and functional attributes of the intestinal microbiota. These omics techniques offer valuable insights into the microbiota’s association with various diseases [17].

While differences in the gut microbiota have been observed between patients with IBD and healthy individuals, the influence of geographic location and nutritional factors across countries is not well understood. Therefore, this study was performed to analyze the bacterial composition of the intestinal microbiota in patients with IBD from Mexico and Spain, considering their dietary and anthropometric data, and to compare these findings with those from healthy controls in both countries.

## 2. Results

In this study, we evaluated the bacterial community of the intestinal microbiota in Mexican and Spanish volunteers with IBD, including UC and CD, and compared it with that in healthy controls. The Mexican cohort comprised 12 participants with UC and 3 with CD (collectively designated MX-IBD), along with 20 healthy controls (MX-H). Similarly, the Spanish cohort comprised 27 participants with IBD (SP-IBD) and 17 healthy controls (SP-H). Dietary and anthropometric assessments were conducted for all the participants.

### 2.1. Anthropometric and Dietary Analysis

An analysis of anthropometric and dietary parameters revealed significant differences between the patients with IBD and the healthy controls in the Mexican and Spanish cohorts (Table 1). The mean weights for the Mexican groups were 69.1 ± 15.2 kg for the MX-IBD group and 60.6 ± 6.1 kg for the MX-H group. In the Spanish groups, the mean weights were 77.8 ± 17.3 kg for the SP-IBD group and 69.6 ± 15.9 kg for the SP-H group (*p* < 0.0015). The Mexican groups exhibited lower fat mass percentages (26.9 ± 5.3% in the MX-IBD group and 24.7 ± 5.6% in the MX-H group) than the Spanish groups (32.6 ± 11.6% in the SP-IBD group and 29.2 ± 4.9% in the SP-H group) (*p* < 0.0334). Conversely, the Mexican participants showed a higher percentage of lean mass (31.3 ± 2.9% in the MX-IBD group and 29.7 ± 5.7% in the MX-H group) than the Spanish participants (24.2 ± 1.3% in the SP-IBD group and 23.7 ± 3.4% in the SP-H group) (*p* < 0.001). Considering the gender by group, the Mexican women with IBD exhibited higher percentages of fat mass (34.5 ± 7.7%) than the female population in the Spanish cohort (32.9 ± 11.9% in the IBD group and 31.8 ± 5.0% in the control group). In the male cohort, the Spanish men demonstrated a higher percentage of fat (32.2 ± 6.8% and 25.7 ± 3.9% for the IBD and control groups, respectively) compared to the Mexican men (20.3 ± 4.9% and 23.9 ± 5.0% for the IBD and control groups, respectively). Regarding the lean mass, the female cohort from the Mexican groups displayed the highest percentages (27.38 ± 3.1% and 29.2 ± 6.2% for the IBD and control groups, respectively) when compared to the female cohort of the Spanish groups (18.7 ± 1.1% and 21.6 ± 3.0% for the IBD and control groups, respectively). In the male cohort, the Mexican groups exhibited the highest percentages of lean mass (32.6 ± 3.3% and 30.7 ± 5.1% for the IBD and control groups, respectively) compared to the male cohort of the Spanish groups (29.5 ± 2.0% and 26.5 ± 4.4% for the IBD and control groups, respectively).

The daily kilocalorie intake was significantly higher in the MX-H group (1580.6 ± 188.8 kcal) than in the other groups (941.1 ± 135.5 kcal for the MX-IBD group, 1471.8 ± 548.0 kcal for the SP-IBD group, and 1347.7 ± 463.5 kcal for the SP-H group) (*p* < 0.0001). Carbohydrates were the primary source of dietary energy for all the groups, comprising 44.2% to 54.1% of the total caloric intake (*p* < 0.0139). The SP-IBD and SP-H groups had the highest carbohydrate intake (54.1 ± 6.7% and 53.7 ± 7.9%, respectively) and the highest intake of complex carbohydrates (49.2 ± 6.7% and 50.7 ± 8.1%, respectively) (*p* < 0.0001). The Mexican groups exhibited a higher intake of proteins and lipids: 21.6 ± 4.0% (MX-IBD) and 22.6 ± 4.9% (MX-H) for proteins (*p* < 0.0359), and 34.1 ± 7.3% (MX-IBD) and 30.8 ± 8.1% (MX-H) for lipids (*p* < 0.0179). The Mexican groups also had a higher percentage of calories from meat products [13.6 ± 3.6% (MX-IBD) and 12.1 ± 3.5% (MX-H)] than the Spanish groups [8.9 ± 2.6% (SP-IBD) and 7.1 ± 2.1% (SP-H)] (*p* > 0.006).

### 2.2. Gut Microbiota Characterization

**Figure 1:**
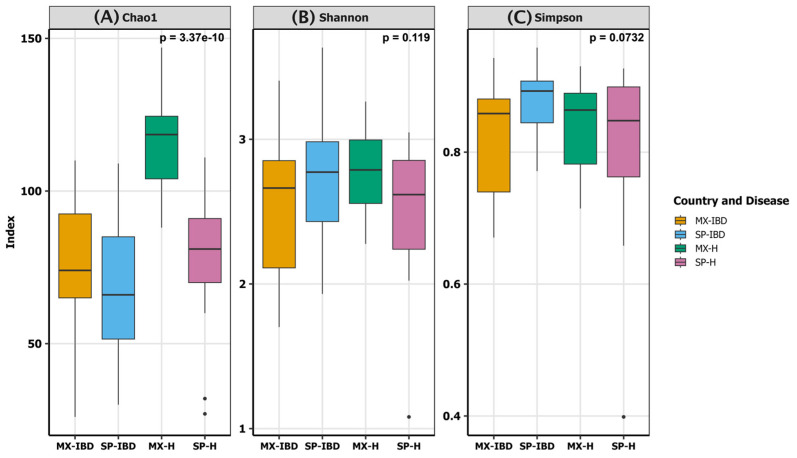
Alpha diversity indices ((**A**): Chao1 for species richness; (**B**): Shannon for diversity; and (**C**): Simpson for evenness) comparing the gut microbiota of the Mexican and Spanish participants with IBD and the healthy controls. MX-IBD, Mexican participants with IBD; MX-H, healthy Mexican participants; SP-IBD, Spanish participants with IBD; SP-H, healthy Spanish participants.

To characterize the intestinal bacterial communities, we performed an amplicon sequencing of the 16S rRNA gene, focusing on the V3–V4 hypervariable regions. In total, 5,373,421 reads were classified using the SILVA reference database. The rarefaction curve indicated that our sequencing depth was sufficient to capture the bacterial diversity within the samples (Figure S1). Among the three alpha diversity indices calculated (Chao1, Shannon, and Simpson) (Figure 1), only the Chao1 richness estimator revealed a statistically significant difference between the groups (*p* = 3.37 × 10^−10^). The MX-H group exhibited the highest Chao1 index, while the SP-IBD group had the lowest (Figure 1A). No significant differences were observed for the Shannon index (*p* = 0.119) or Simpson index (*p* = 0.0732). The Shannon index accounts for species richness and evenness, while the Simpson index emphasizes evenness. The SP-IBD and MX-H groups displayed the highest Shannon values, while the SP-IBD and SP-H groups showed the highest Simpson values (Figure 1B,C). Despite the lower species richness in the SP-IBD group compared to the MX-IBD group, as indicated by the Chao1 index, the Shannon and Simpson indices were higher in the SP-IBD group. This suggests that while IBD may be associated with reduced richness, it could also lead to a more even distribution of the remaining taxa, particularly in the Spanish cohort. Additionally, the MX-H group demonstrated a higher richness (Chao1) and diversity (Shannon) than the SP-H group, but a lower evenness (Simpson). These findings highlight the complex interplay between disease status, nationality, and the multifaceted nature of gut microbial alpha diversity.

**Figure 2:**
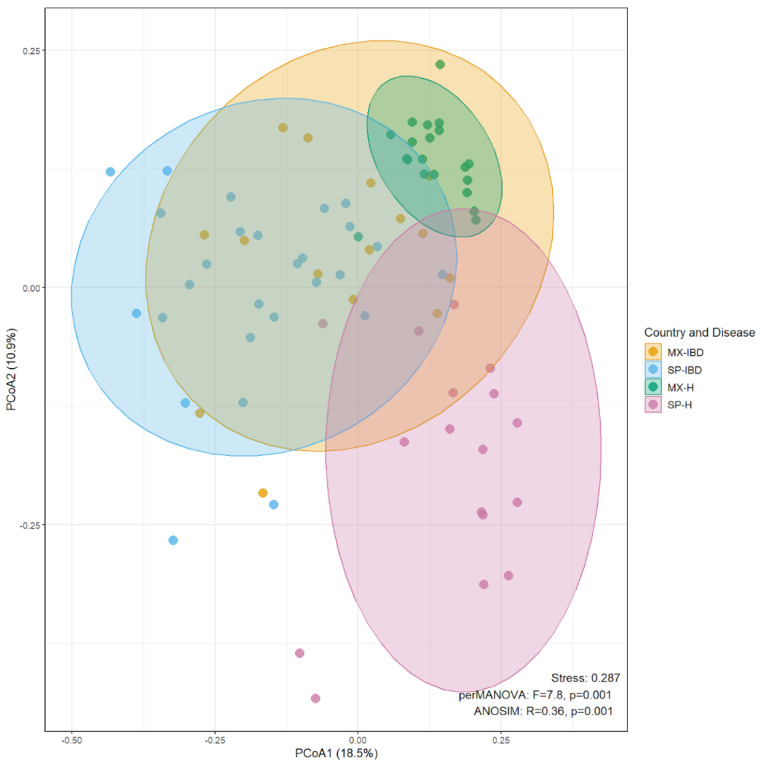
PCoA plots based on Bray–Curtis distances, showing the genus-level gut microbiota profiles of the Mexican and Spanish individuals with IBD and the healthy controls. The figure illustrates the microbial community structure and its variation across the countries and disease statuses. MX-IBD, Mexican participants with IBD; MX-H, healthy Mexican participants; SP-IBD, Spanish participants with IBD; SP-H, healthy Spanish participants.

Principal coordinates analysis (PCoA) based on Bray–Curtis dissimilarities revealed distinct clustering patterns in the gut microbiota composition among the four groups (MX-IBD, SP-IBD, MX-H, and SP-H) (Figure 2). Both a permutational multivariate analysis of variance (perMANOVA) and analysis of similarities (ANOSIM) confirmed significant differences in the beta diversity between the groups (*p* = 0.001 for both analyses). Pairwise perMANOVA comparisons further underscored these differences, with all the pairwise comparisons showing statistically significant variations in the beta diversity (*p* < 0.001) (Table S2). The PCoA ellipses showed that the MX-H ellipse was entirely contained within the MX-IBD ellipse, suggesting that the IBD group encompassed a broader range of bacterial communities, including those typical of the healthy group. Overlap was observed between the SP-IBD and MX-IBD ellipses, indicating shared bacterial communities, while the MX-H ellipse exhibited a minimal overlap with the SP-IBD ellipse. Additionally, the MX-H and SP-H ellipses displayed a limited overlap, suggesting distinct bacterial community structures among the healthy individuals from different nationalities. Overall, these findings indicate that while IBD is associated with alterations in gut microbial community structures, considerable inter-individual variability exists within both healthy and IBD groups. Moreover, nationality appears to influence gut microbial communities, even in the context of disease.

**Figure 3:**
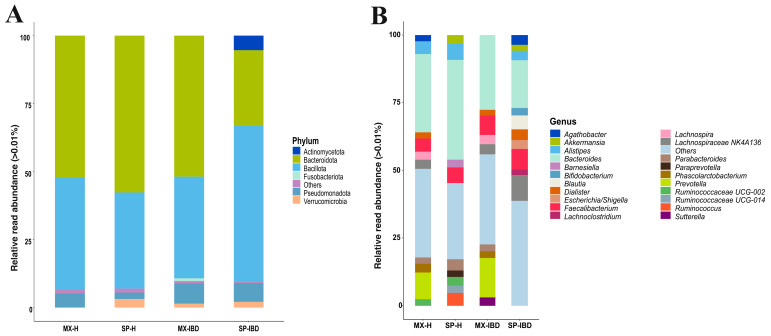
Bar plot showing the bacterial composition of the gut microbiota in the Mexican and Spanish individuals with IBD and the healthy controls, displayed at the (**A**) phylum level and (**B**) genus level. MX-IBD, Mexican participants with IBD; MX-H, healthy Mexican participants; SP-IBD, Spanish participants with IBD; SP-H, healthy Spanish participants.

The taxonomic composition of the gut microbiota at the phylum level revealed that two bacterial phyla—Bacteroidota (formerly Bacteroidetes) and Bacillota (formerly Firmicutes)—dominated across all four groups (Figure 3A). Bacteroidota was the most abundant phylum in the MX-H, SP-H, and MX-IBD groups, comprising 52.07%, 57.68%, and 51.83% of the relative abundance, respectively (Table S3). However, in the SP-IBD group, Bacteroidota was the second most abundant phylum, with a relative abundance of 27.70%. Conversely, Bacillota exhibited relative abundances of 41.45%, 35.36%, 37.44%, and 57.51% in the MX-H, SP-H, MX-IBD, and SP-IBD groups, respectively. The phyla Pseudomonadota (formerly Proteobacteria), Verrucomicrobiota (formerly Verrucomicrobia), Fusobacteriota (formerly Fusobacteria), and Actinomycetota exhibited relative abundances ranging from 1.02% to 7.49%. Fusobacteriota was detected only in the MX-IBD group, while Verrucomicrobiota was absent in the MX-H group. Actinomycetota was present only in the SP-IBD group.

At the genus level, *Bacteroides* was the most abundant taxon across all four groups, with relative abundances ranging from 17.58% to 36.78%, followed by *Prevotella* (9.80% to 14.45%) and *Faecalibacterium* (4.88% to 7.66%). Notably, *Prevotella* was observed only in the Mexican groups, with a higher abundance in the MX-IBD group (Figure 3B and Table S4). Several genera exhibited distinct patterns of the relative abundance by group. *Akkermansia* was detected only in the Spanish groups, with a higher relative abundance in the SP-IBD group (2.09% to 3.09%). By contrast, *Bifidobacterium*, *Blautia*, *Escherichia/Shigella*, and *Lachnoclostridium* were found exclusively in the SP-IBD group, with relative abundances of 2.78%, 5.11%, 3.24%, and 2.14%, respectively. *Paraprevotella*, *Ruminococcaceae UCG-014 5*, and *Ruminococcus* were present only in the SP-H group, with relative abundances of 2.54%, 2.73%, and 4.75%, respectively. *Sutterella* (3.19%) was uniquely observed in the MX-IBD group. The genera uniquely present in the IBD groups included *Escherichia/Shigella*, *Blautia*, *Bifidobacterium*, and *Lachnoclostridium* in the SP-IBD group and *Sutterella* in the MX-IBD group. These genera were absent in the healthy individuals of both nationalities.

### 2.3. Interplay Between Microbiota, Host Factors, and Diet

**Figure 4:**
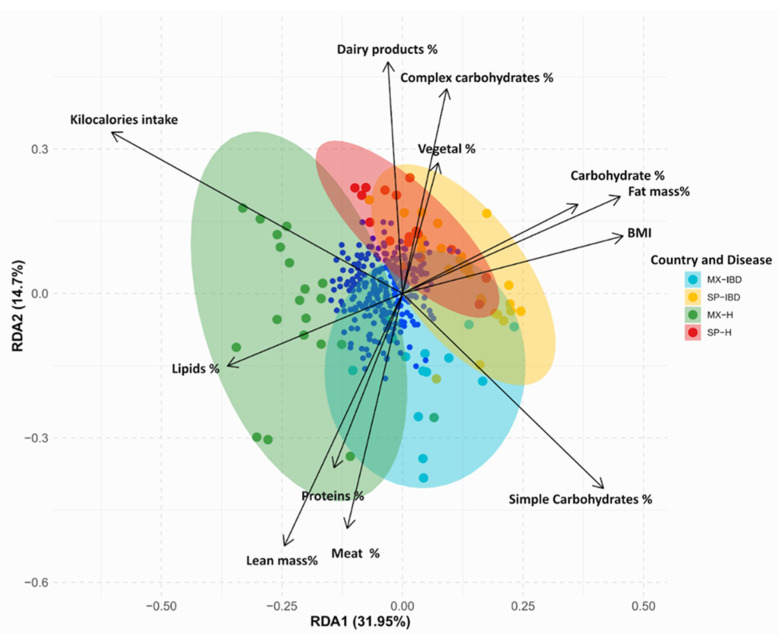
Biplot of the RDA showing the relationships between the gut bacterial community composition and anthropometric factors (e.g., lean mass, fat mass) and dietary factors (e.g., kilocalories, protein intake) in the Mexican and Spanish participants with IBD and the healthy controls. MX-IBD, Mexican participants with IBD; MX-H, healthy Mexican participants; SP-IBD, Spanish participants with IBD; SP-H, healthy Spanish participants.

Redundancy analysis (RDA) was used to explore the relationships between the gut bacterial community composition and anthropometric and dietary parameters (Figure 4). The analysis revealed distinct patterns of association among the four study groups. In the MX-IBD group, the gut bacterial community composition was significantly correlated with the simple carbohydrates, meat, and lean mass. In the SP-IBD and SP-H groups, the fat mass and dairy product consumption were the most influential factors. For the MX-H group, the kilocalorie intake and lean mass were significantly associated with the intestinal microbiota. The first three components of the RDA explained 56% of the variability in the gut bacterial community composition (RDA1: 31.95%; RDA2: 14.70%; RDA3: 9.34%) (Table S5). The kilocalorie intake was negatively correlated with the RDA1, while the body mass index (BMI), simple carbohydrates, and fat mass showed positive correlations. In the RDA2, the meat and lean mass were negatively correlated, whereas the complex carbohydrates and dairy products exhibited positive correlations (Table S6).

**Figure 5:**
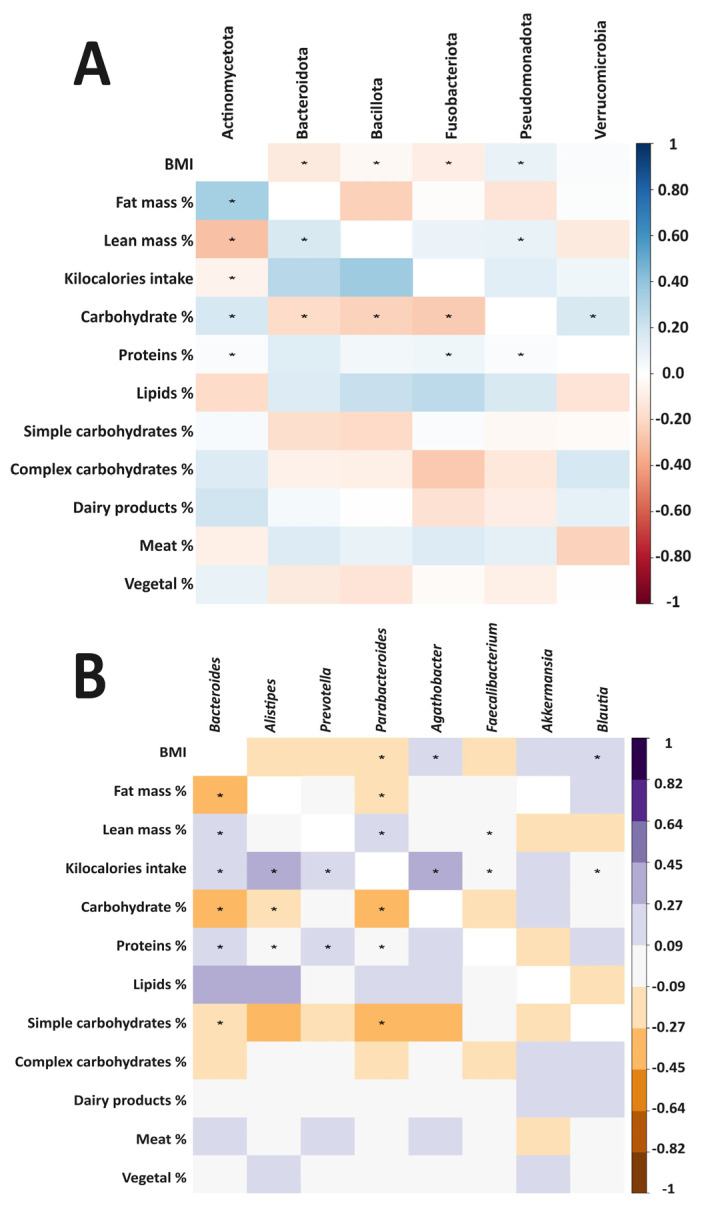
Spearman correlation plot showing the relationships between the intestinal microbiota and anthropometric and dietary parameters in the participants with IBD and the healthy controls from Mexico and Spain. (**A**) Phylum level. (**B**) Genus level. *p* < 0.05 *.

A corrplot was generated using Pearson correlation analysis to identify and visualize the relationships between the relative abundances of the most prevalent bacterial phyla and genera and selected anthropometric and dietary parameters (Figure 5). Several significant positive and negative correlations (*p* < 0.05) were observed at both the phylum and genus levels. At the phylum level, the BMI was positively correlated with the Pseudomonadota and negatively correlated with the Bacteroidota, Bacillota, and Fusobacteria. The fat mass was positively correlated with the Actinomycetota, while lean mass showed positive correlations with the Bacteroidota and Pseudomonadota and a negative correlation with the Actinomycetota (Figure 5A). The kilocalorie intake was negatively correlated with the Actinomycetota, while the dietary carbohydrates showed positive correlations with the Actinomycetota and Verrucomicrobia and negative correlations with the Bacteroidota, Bacillota, and Fusobacteria. The protein intake was positively correlated with the Actinomycetota, Fusobacteria, and Pseudomonadota.

At the genus level, the BMI showed positive correlations with the *Agathobacter* and *Blautia* and a negative correlation with the *Parabacteroides* (Figure 5B). The fat mass was negatively correlated with the *Bacteroides* and *Parabacteroides*, while the lean mass showed positive correlations with the *Bacteroides*, *Parabacteroides*, and *Faecalibacterium*. The kilocalorie intake was positively correlated with the *Bacteroides*, *Alistipes*, *Prevotella*, *Agathobacter*, *Faecalibacterium*, and *Blautia*. The carbohydrate intake showed negative correlations with the *Bacteroides*, *Alistipes*, and *Parabacteroides*, while the protein intake was positively correlated with the *Bacteroides*, *Alistipes*, *Prevotella*, and *Parabacteroides*. Finally, the simple carbohydrates were negatively correlated with the *Bacteroides* and *Parabacteroides*.

## 3. Discussion

In this study, we used molecular techniques to analyze the intestinal microbiota in individuals with IBD from Mexico and Spain as well as the healthy controls from both countries. Additionally, we explored correlations between these microbiota findings and the anthropometric and dietary parameters.

Reduced biodiversity in the intestinal microbiota is now recognized as a key factor in the development of IBD [18]. Our study showed that microbial richness, as measured by the Chao1 index, was higher in the MX-H group and lower in the SP-IBD group. These findings align with previous research documenting the decreased microbial diversity in patients with IBD. For instance, Aldars-García et al. (2021) reported that the intestinal microbiome in individuals with IBD is generally characterized by reduced species richness and diversity [10]. However, some studies have shown that the low diversity and richness in the fecal microbiota of individuals with IBD can be partially restored. For example, one study demonstrated a significant increase in the intestinal microbiota richness and diversity in patients with CD following treatment with infliximab, a drug used to alleviate symptoms of CD and UC [19]. Another study showed that enteral nutrition in pediatric patients with CD led to increased microbial diversity, as measured by the Shannon and Simpson indices [20]. This association between IBD and reduced bacterial diversity may be linked to the dysbiosis commonly observed in these conditions [8].

Significant differences in the community composition (beta diversity) were observed between the cohorts, indicating variability in the bacterial communities between the participants with IBD and the healthy controls as well as between the nationalities. These findings suggest that, while IBD is associated with alterations in intestinal bacterial community structures, interindividual variability may be influenced by both the presence of IBD [21] and geographical location [22]. Our results show that the phyla Bacteroidota and Bacillota dominated both the IBD and healthy groups across the nationalities, although their relative abundances varied. Similarly, Tian et al. reported that Bacteroidota and Bacillota dominate the gut microbiota, comprising up to 98% of the total gut bacteria [23]. The observed changes in the proportions of Bacteroidota and Bacillota may be attributed to the depletion of these phyla and the increased abundance of Pseudomonadota and Actinomycetota in individuals with IBD [18,24]. This finding is consistent with our results, as the presence of Pseudomonadota in the Spanish IBD group was primarily due to the *Escherichia/Shigella*, while in the Mexican IBD group, it was attributed to the *Sutterella*. Similarly, the increase in Actinomycetota in the Spanish participants with IBD was associated with the presence of *Bifidobacterium*. The high abundance of Bacillota in the Spanish IBD group was characterized by the specific bacterial genera known to produce short-chain fatty acids (SCFAs), including *Agathobacter*, *Blautia* [25], Lachnospiraceae NK4A136, and *Lachnoclostridium* [26].

Diet is a key environmental factor that significantly influences the composition of the intestinal microbiota [27]. For example, evidence has shown that the Mediterranean diet is associated with a lower prevalence of *Prevotella* in the intestinal microbiota [28], while a diet rich in complex carbohydrates has been linked to an increased presence of *Bacteroides* [29]. These findings align with our study, in which both Spanish groups, known for their adherence to the Mediterranean diet, exhibited an absence of *Prevotella*. Furthermore, the positive correlation between the *Prevotella* and lean mass observed in this study is consistent with a previous study evaluating the intestinal microbiota in Mexican people with a healthy weight and obesity. That study reported a relationship between *Prevotella* and parameters associated with healthy weight, such as lean mass [9]. Additionally, a higher intake of complex carbohydrates was observed, with the healthy Spanish group showing a greater presence of *Bacteroides*.

Although several studies have revealed a decrease in the genus *Faecalibacterium* in individuals with IBD [30,31,32,33], our results revealed a higher prevalence of this genus in the IBD groups of both nationalities than in the healthy controls. This finding may be related to evidence suggesting that a high abundance of *Faecalibacterium*, particularly *Faecalibacterium prausnitzii*, is associated with protection against IBD because of its production of butyric acid [34]. Additionally, other studies have shown that *Faecalibacterium* can protect the host from mucosal inflammation through mechanisms such as the downregulation of inflammatory cytokines [35] or the stimulation of interleukin-10, a cytokine with anti-inflammatory properties [36].

Similar to the *Faecalibacterium*, the presence of *Bifidobacterium* in the Spanish IBD group may be related to its beneficial health effects, such as enhancing mucosal barrier function and reducing inflammation through the production of SCFAs and vitamins [37]. Additionally, strains of *Bifidobacterium*, such as *B. longum* and *B. breve*, are used as probiotics in alternative treatments for IBD [38]. One study revealed that members of *Bifidobacterium* exert a suppressive effect on intestinal pathogens, including *Bacteroides vulgatus*, a bacterium associated with IBD pathogenesis [39]. Although the presence of *Faecalibacterium* and *Bifidobacterium* might seem beneficial based on the current literature, this observation is preliminary, and further studies are needed to explore the role of these bacteria in IBD.

The presence of *Prevotella* exclusively in the Mexican groups and *Akkermansia* exclusively in the Spanish groups could be attributed to multiple factors, such as the geographical location, dietary habits, and genetic variations between the populations, which may influence the composition and function of the intestinal microbiota [40]. However, there is still insufficient information on the role of *Prevotella* and *Akkermansia* in IBD. Although *Akkermansia muciniphila* has been linked to several health benefits, including improved intestinal barrier function and reduced inflammation [41], further research is necessary to elucidate its potential contributions to disease pathogenesis and explore its therapeutic implications.

Complex carbohydrates, particularly dietary fiber, are fermented by the gut microbiota [42]. This leads to the production of SCFAs such as butyrate, which exert anti-inflammatory effects on the intestinal lining. Fiber-rich diets have been shown to support the growth of beneficial bacteria, such as *Bifidobacterium*, which are often depleted in individuals with IBD [14]. Conversely, low-fiber diets may reduce SCFA production, potentially worsening intestinal inflammation [15]. High-protein diets, particularly those rich in animal protein, have been linked to the production of harmful metabolites such as hydrogen sulfide and ammonia, which can exacerbate inflammation and damage the intestinal epithelium [43]. Moreover, certain dietary fats have been associated with dysbiosis—an imbalance in the gut microbiota composition—and are negatively correlated with overall health outcomes [9].

Collectively, this body of evidence highlights the critical role of the diet in modulating the intestinal microbiota and influencing the progression of IBD. Future research should aim to further delineate the specific effects of individual macronutrients, as well as their quantities, on the gut microbiota in IBD, and their impact on both clinical manifestations and symptoms. Such research will provide valuable insights into the mechanisms by which dietary interventions may influence disease pathogenesis.

We acknowledge two main limitations in our study that warrant consideration in future research. First, although the sample size is consistent with previous investigations into the gut microbiota in IBD, larger cohorts would strengthen the validity of our conclusions, particularly regarding the associations between the microbial composition, dietary components, and anthropometric parameters. Second, while this study focused primarily on bacterial communities, incorporating other microbial components, such as fungi and viruses, could provide a more comprehensive understanding of the intestinal ecosystem and its implications for IBD.

## 4. Materials and Methods

### 4.1. Participant Recruitment and Sample Collection

This international study recruited participants from Mexico and Spain. The study protocol was approved by the Institutional Review Board at the Instituto de Seguridad y Servicios Sociales de los Trabajadores del Estado (ISSSTE) in Mexico. In Spain, approvals were obtained from the Vall d’Hebrón University Hospital Research Ethics Committee and the Terrassa Hospital Research Ethics Committee. The control participants were required to be Caucasian, be aged 18–60 years, have not used antibiotics in the previous 2 months, follow an omnivorous diet, and be clinically healthy. The patients were required to be Caucasian, be aged 25–65 years, have a BMI of 20.0 to 35.0 kg/m^2^, and be free of underlying pathologies except those associated with excess weight or IBD [44].

### 4.2. Dietary Assessment

To evaluate the usual dietary intake of the participants, we utilized two quantitative questionnaires: (a) The food frequency questionnaire (FFQ) collects information about food consumption over an extended period (1 month). The participants reported the frequency and quantity of their typical intake of each listed item. (b) The 24-h dietary recall (24-HDR) captures detailed information about food intake within a specific 24 h period. All the dietary interviews were conducted by registered dietitians [42].

### 4.3. Anthropometric Evaluation

An anthropometric evaluation was conducted to assess the body composition, including muscle and fat mass. Certified International Society for the Advancement of Kinanthropometry (ISAK) anthropometrists used calibrated instruments: a 0.5 cm-wide metal measuring tape (Cescorf) graduated in millimeters, a digital scale (TANITA UM-040, Tanita Corporation, Tokyo, Japan) with a 150 kg capacity and 100 g readability, and a skinfold caliper (Slim Guide, Creative Health Products, Ann Arbor, MI, USA) graduated in millimeters. The following anthropometric measurements were recorded: weight (kg); height (cm); skinfold thickness (mm) at the triceps, biceps, iliac crest, and subscapular sites; and circumferences (cm) of the hip, waist, arm, and wrist [9].

### 4.4. Microbiota Analysis

#### 4.4.1. Fecal Sample Collection and Processing

Fecal samples were collected from all 79 study participants: 15 patients with IBD from Mexico (MX-IBD), 20 healthy participants from Mexico (MX-H), 27 patients with IBD from Spain (SP-IBD), and 17 healthy participants from Spain (SP-H). The participants received detailed instructions on the proper collection procedures and were provided with sterile containers to store the samples at 4 °C, ensuring sample integrity during their transport to the laboratory. Upon arrival, the samples were frozen at −80 °C until their further processing [9]. For the DNA extraction, 180 to 220 mg aliquots of each frozen fecal sample were processed using the QIAamp^®^ DNA Stool Mini Kit (Hilden, Germany), following the manufacturer’s protocol.

#### 4.4.2. Library Construction and Sequencing

The bacterial taxonomic identification was based on sequences derived from the V3 and V4 hypervariable regions of the 16S rDNA gene. The library construction followed the Illumina protocol for 16S Metagenomic Sequencing Library Preparation. The microbial genomic DNA was stored at a concentration of 5 ng/μL in 10 mM Tris buffer (pH 8.5) and quantified using a Qubit fluorometer (Thermo Fisher Scientific, Waltham, MA, USA). The DNA was then amplified using specific primers targeting the V3–V4 regions of the 16S rRNA gene [45]. Adapter ligation for multiplexing was performed using the Nextera XT Index Kit (FC-131-1096), incorporating indices to distinguish the individual samples. Before the sequencing, the library was re-quantified with the Qubit fluorometer. The sequencing was performed with a MiSeq sequencer using the MiSeq v3 reagent kit (MS-102-3001) with 2 × 300 bp paired-end reads, according to Illumina’s guidelines. The primers used were as follows: the forward primer 5′-TCGTCGGCAGCGTCAGATGTGTATAAGAGACAGCCTACGGGNGGCWGCAG-3′ and reverse primer 5′-GTCTCGTGGGCTCGGAGATGTGTATAAGAGACAGGACTACHVGGGTATCTAATCC-3′ [46].

#### 4.4.3. Bioinformatic Analysis

The bacterial composition of the gut microbiota, including the relative abundance and ecological diversity, was analyzed using QIIME 2.0 software (Quantitative Insights Into Microbial Ecology) [47]. The DADA2 algorithm was employed to filter chimeric sequences, correct errors, and remove noisy reads, resulting in accurate amplicon sequence variants [48]. Denoising was applied to paired-end reads using 8 threads, with sequences truncated at 250 bp (forward) and 220 bp (reverse) and trimmed at 17 bp (forward) and 21 bp (reverse). Feature tables (FeatureData(Sequence) and FeatureData(Taxonomy)) were generated with a 99% identity threshold against the SILVA 132 16S rRNA gene reference database [49].

Human sequences were filtered out using Bowtie2 (version 2.3.4.2) against the GRCh38.p13 human reference database before the taxonomic assignment. The q2-feature-classifier plugin was used to assign a taxonomy to the representative sequences via a trained Naive Bayes classifier. The taxonomic classification was performed using the classify–sklearn method, and the designated sequences were archived along with the trained classifier. The taxonomic data were linked to the amplicon sequence variant table, and taxon bar charts were exported in CSV format from QIIME 2 View (view.qiime2.org, accessed on 10 August 2024).

### 4.5. Statistical Analysis

Anthropometric and dietary variables were compared between the study groups using ANOVA. Pairwise t-tests with the Bonferroni adjustment were applied to account for multiple comparisons, with a significance level of *p* < 0.05 considered statistically significant. These analyses were performed using RStudio (R version 4.4.1) [50]. The ANOVA was conducted with the aov() function, and pairwise comparisons were performed using the pairwise.t.test() function with the Bonferroni adjustment.

For the microbial community analyses, downstream analyses were conducted at the genus taxonomic level, except for the relative abundance analysis, which was performed at both the phylum and genus levels. Statistical analyses and data visualization were performed using RStudio and the ggplot2 package (version 3.3.3) [51]. The DESeq2 package was used to normalize read counts from the 16S rRNA gene sequences [52]. Rarefaction analysis, to account for variations in sequencing depth, was performed using the rarecurve() function from the vegan package [9].

The alpha diversity metrics, including Shannon’s diversity index, Simpson’s diversity index, and the Chao1 richness estimator, were calculated using the diversity() and estimateR() functions from the vegan package [9]. Box plots were used to visualize these metrics, grouped by the country and disease status. Relative abundance analysis was conducted at both the phylum and genus levels, with taxa having a mean relative abundance below 0.01% consolidated into an “Others” category. Bar plots were used to depict the relative abundance of the most prevalent phyla and genera across the four groups (MX-IBD, SP-IBD, MX-H, and SP-H).

PCoA was used to explore the patterns of beta diversity, with visualization according to the country and disease status. The Bray–Curtis dissimilarity was used to quantify differences between samples, calculated with the vegdist() function from the vegan package [53]. Differences in the community composition between the groups were assessed with perMANOVA (*p* < 0.05) and ANOSIM, using the adonis2() and anosim() functions from the vegan package [54].

RDA was performed to explore the relationship between the microbial community composition and relevant host factors, including anthropometric parameters (BMI, fat mass %, and lean mass %) and dietary intake parameters (kilocalories, carbohydrates %, proteins %, lipids %, simple carbohydrates %, complex carbohydrates %, dairy products %, meat %, and vegetables %). The RDA was conducted using the rda() function from the vegan package [55], with the significance of the RDA axes assessed to understand the influence of these parameters on the microbial community composition, considering the potential impact of the country and disease status.

Biplots were generated using the ggord package to visualize the relationships between the samples, bacterial genera, and selected parameters in the RDA ordination space [56]. Pearson correlation analysis was used to evaluate the relationships between the relative abundances of the most prevalent bacterial genera and selected anthropometric and dietary intake parameters, accounting for variations across the country and disease groups. The cor() function computed the Pearson correlation coefficients [57], and the cor.mtest() function from the corrplot package was used to assess the statistical significance of the correlations, with a threshold of *p* < 0.05 [58]. A correlation plot was generated to visualize the strength and direction of these associations.

## 5. Conclusions

This study identified reduced microbial richness as measured by the Chao1 index in the individuals with IBD, particularly within the Spanish cohort. Significant differences in the bacterial composition of the intestinal microbiota were observed between the Mexican and Spanish participants with IBD. The most abundant phyla across all groups were Bacteroidota and Bacillota. *Prevotella* was found exclusively in the Mexican groups, while *Akkermansia* was present only in the Spanish groups. *Faecalibacterium* was highly prevalent in the IBD groups, and *Bifidobacterium* was found only in the Spanish IBD group. The dietary patterns observed in this study suggest that specific bacteria within the gut microbiota can significantly influence both the abundance and diversity of gut bacteria, highlighting the critical role of nutrition in modulating the intestinal microbiome in the context of IBD. These findings enhance our understanding of the gut microbiota’s role in IBD and underscore the need to consider geographic and dietary influences in future research and treatment strategies.

## Figures and Tables

**Table 1:** Sociodemographic, anthropometric, and dietary analysis of the four study groups.

Characteristics	Groups of Participants				*p* Value
	MX-IBD	SP-IBD	MX-H	SP-H	
n	15	27	20	17	
Gender (female/male)	8/7	10/17	12/8	9/8	
Weight (kg)	69.1 ± 15.2	77.8 ± 17.3	60.6 ± 6.1	69.6 ± 15.9	0.0015 *
Female	65.4 ± 14.6	65.2 ± 15.1	58.12 ± 4.9	62.9 ± 13.2	0.0417 *
Male	73.2 ± 15.6	83.3 ± 21.0	64.35 ± 6.3	78.1 ± 16.6	0.0139 *
Fat mass %	26.9 ± 5.3	32.6 ± 11.6	24.7 ± 5.6	29.2 ± 4.9	0.0334 *
Female	34.5 ± 7.7	32.9 ± 11.9	24.22 ± 5.3	31.8 ± 5.0	0.0250 *
Male	20.3 ± 4.9	32.2 ± 6.8	23.90 ± 5.0	25.7 ± 3.9	0.0389 *
Lean mass %	31.3 ± 2.9	24.2 ± 1.3	29.7 ± 5.7	23.7 ± 3.4	0.0001 *
Female	27.38 ± 3.1	18.7 ± 1.1	29.2 ± 6.2	21.6 ± 3.0	0.0200 *
Male	32.6 ± 3.3	29.5 ± 2.0	30.70 ± 5.1	26.5 ± 4.4	0.0567
Daily caloric intake (kcals)	941.1 ± 135.5	1471.8 ± 548.03	1580.6 ± 188.8	1347.7 ± 463.5	0.0001 *
Carbohydrates %	44.2 ± 19.0	54.1 ± 6.7	46.4 ± 9.9	53.7 ± 7.9	0.0139 *
Proteins %	21.6 ± 4.0	19.8 ± 4.3	22.6 ± 4.9	17.6 ± 3.2	0.0359 *
Lipids %	34.1 ± 7.3	24.3 ± 6.6	30.8 ± 8.1	25.7 ± 6.5	0.0179 *
Simple carbohydrates %	11.3 ± 14.3	4.8 ± 4.7	1 ± 2.0	3.02 ± 3.8	0.0005 *
Complex carbohydrates %	32.8 ± 6.1	49.2 ± 6.7	45.4 ± 7.7	50.7 ± 8.1	0.0001 *
Dairy products %	1.8 ± 0.4	4.8 ± 1.2	4.05 ± 1.7	3.8 ± 1.3	0.0097 *
Meat %	13.6 ± 3.6	8.9 ± 2.6	12.12 ± 3.5	7.1 ± 2.05	0.006 *
Vegetal %	5.2 ± 1.2	6.0 ± 1.01	5.9 ± 1.1	6.7 ± 1.5	0.4855

Values are presented as mean ± standard deviation. *p*-values were calculated using ANOVA to assess overall group differences. * Statistically significant results are indicated by *p* < 0.05. Refer to Table S1 for a detailed pairwise comparison. MX-IBD, Mexican participants with IBD; MX-H, healthy Mexican participants; SP-IBD, Spanish participants with IBD; SP-H, healthy Spanish participants.

## Data Availability

The sequence data for the 16S rRNA gene have been deposited in the EBI Short Read Archive repository (https://www.ebi.ac.uk/ena, accessed on 15 August 2023) under the study accession number PRJEB35085 with accession numbers ERS3922721 to ERS3922799.

## Supplementary Materials

The supporting information can be downloaded at: https://www.mdpi.com/article/10.3390/ijms252211887/s1.
