# Supplementary material for: Gut Bacterial Composition and Nutritional Implications in Mexican and Spanish Individuals with Inflammatory Bowel Disease Compared to Healthy Controls
Source: Int J Mol Sci. 2024 Nov 5;25(22):11887. doi: 10.3390/ijms252211887 (PMC11593679; doi:10.3390/ijms252211887)
Supplement: Supplementary file 1 [file ijms-25-11887-s001.zip › ijms-3216732-supplementary.pdf]

Supplementary Material for

Gut Bacterial Composition and Nutritional Implications in Mexican and Spanish Individuals with Inflammatory Bowel Disease Compared to Healthy Controls

Figure S1. Rarefaction curve showing sampling depth and the number of genera observed.

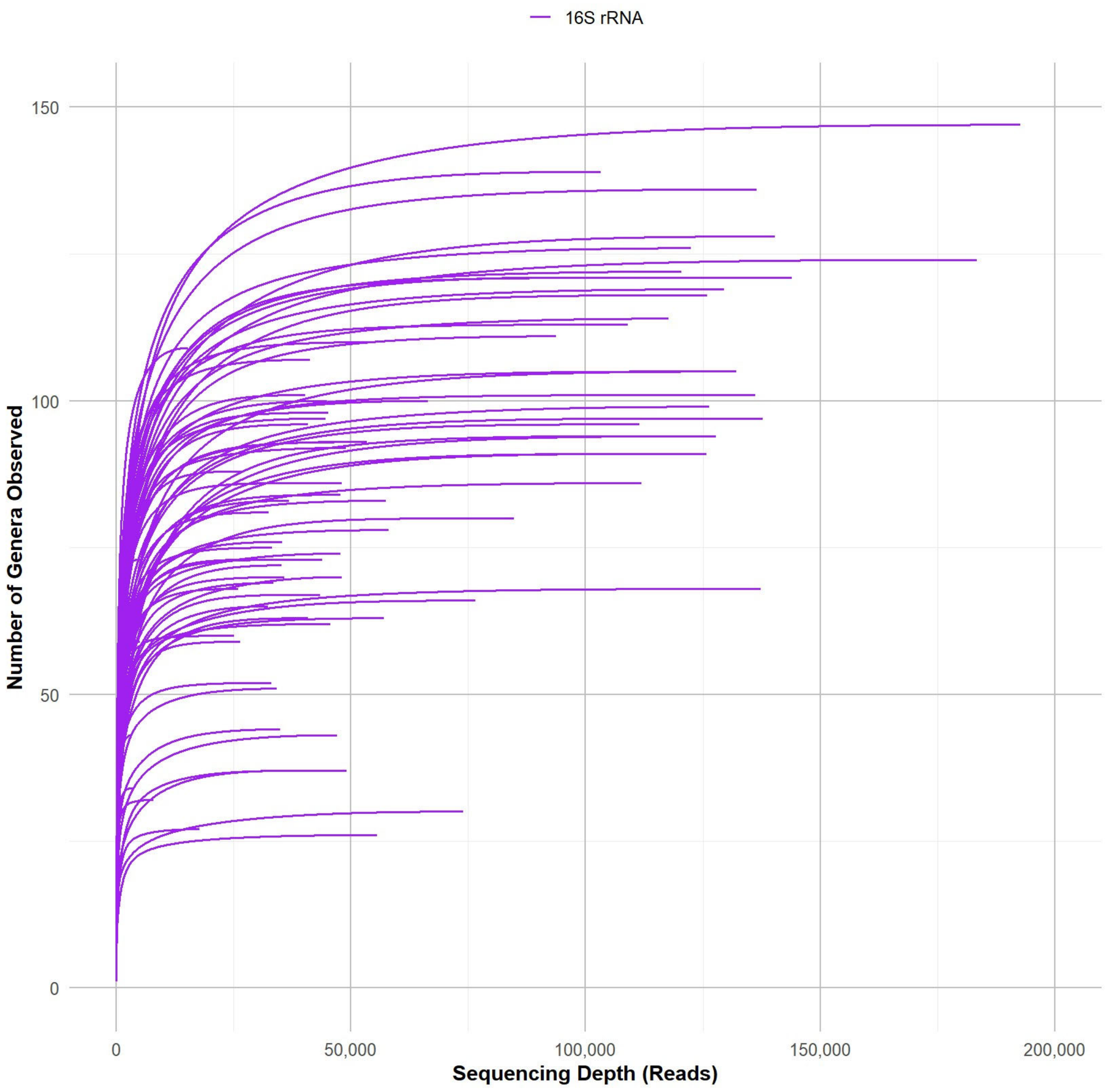

Table S1. Pairwise comparisons of anthropometric and dietary variables between Mexican and Spanish participants with inflammatory bowel disease (MX-IBD, SP-IBD) and healthy controls (MX-H, SP-H). P-values were calculated using ANOVA with post-hoc pairwise comparisons. P-values in **bold** indicate statistical significance at the 0.05 level.

Variable	Groups	P-value
**Lean mass %**	MX-IBD vs. MX-H	**2.35E-11**
	SP-H vs. MX-H	1
	SP-IBD vs. MX-H	**0.00672792**
	SP-H vs. MX-IBD	**1.01E-12**
	SP-IBD vs. MX-IBD	**8.96E-07**
	SP-IBD vs. SP-H	**0.00017016**
**Fat mass %**	MX-IBD vs. MX-H	1
	SP-H vs. MX-H	1
	SP-IBD vs. MX-H	**0.03055226**
	SP-H vs. MX-IBD	1
	SP-IBD vs. MX-IBD	0.36523659
	SP-IBD vs. SP-H	1
**Weight (kg)**	MX-IBD vs. MX-H	0.56196909
	SP-H vs. MX-H	0.4265107
	SP-IBD vs. MX-H	**0.00064248**
	SP-H vs. MX-IBD	1
	SP-IBD vs. MX-IBD	0.37055765
	SP-IBD vs. SP-H	0.430149
**Daily caloric intake (Kcals)**	MX-IBD vs. MX-H	**1.92E-05**
	SP-H vs. MX-H	0.6673021
	SP-IBD vs. MX-H	1
	SP-H vs. MX-IBD	**0.00961256**
	SP-IBD vs. MX-IBD	**8.77E-05**
	SP-IBD vs. SP-H	1
**Carbohydrate %**	MX-IBD vs. MX-H	1
	SP-H vs. MX-H	0.36033795
	SP-IBD vs. MX-H	0.1366837
	SP-H vs. MX-IBD	0.13756962
	SP-IBD vs. MX-IBD	**0.04643357**
	SP-IBD vs. SP-H	1
**Proteins %**	MX-IBD vs. MX-H	1
	SP-H vs. MX-H	**0.03967413**
	SP-IBD vs. MX-H	0.41391294
	SP-H vs. MX-IBD	0.24326789
	SP-IBD vs. MX-IBD	1
	SP-IBD vs. SP-H	1
**Lipids %**	MX-IBD vs. MX-H	1

	SP-H vs. MX-H	0.92840896
	SP-IBD vs. MX-H	0.22412782
	SP-H vs. MX-IBD	0.18524516
	SP-IBD vs. MX-IBD	**0.02842637**
	SP-IBD vs. SP-H	1
**Simple carbohydrates %**	MX-IBD vs. MX-H	**0.00030762**
	SP-H vs. MX-H	1
	SP-IBD vs. MX-H	0.3585875
	SP-H vs. MX-IBD	**0.00943387**
	SP-IBD vs. MX-IBD	**0.03001599**
	SP-IBD vs. SP-H	1
**Complex carbohydrates %**	MX-IBD vs. MX-H	0.00697138
	SP-H vs. MX-H	0.936629
	SP-IBD vs. MX-H	1
	SP-H vs. MX-IBD	0.00012108
	SP-IBD vs. MX-IBD	6.05E-05
	SP-IBD vs. SP-H	1
**Dairy products %**	MX-IBD vs. MX-H	0.12746053
	SP-H vs. MX-H	1
	SP-IBD vs. MX-H	1
	SP-H vs. MX-IBD	0.28534599
	SP-IBD vs. MX-IBD	0.00489445
	SP-IBD vs. SP-H	1
**Meat %**	MX-IBD vs. MX-H	1
	SP-H vs. MX-H	0.07341259
	SP-IBD vs. MX-H	0.38087854
	SP-H vs. MX-IBD	0.01418488
	SP-IBD vs. MX-IBD	0.07236984
	SP-IBD vs. SP-H	1
**Vegetal %**	MX-IBD vs. MX-H	1
	SP-H vs. MX-H	1
	SP-IBD vs. MX-H	1
	SP-H vs. MX-IBD	0.72984054
	SP-IBD vs. MX-IBD	1
	SP-IBD vs. SP-H	1

Table S2. Statistical significance of gut microbiota differences (perMANOVA) at the genus level in healthy and IBD Mexican and Spanish subjects.

**Country and Disease**	**perMANOVA (P-value)**
**MX-H vs SP-H**	0.001
**MX-H vs MX-IBD**	0.001
**SP-H vs SP-IBD**	0.001
**MX-IBD vs SP-IBD**	0.001

Table S3. Relative abundance of gut bacterial communities (≥ 0.01%) at the phylum level in healthy and IBD Mexican and Spanish subjects.

Phylum	% Relative read abundance MX-H	% Relative read abundance SP-H	% Relative read abundance MX-IBD	%Relative read abundance SP-IBD
Bacteroidota	52.07	57.68	51.83	27.70
Bacillota	41.45	35.36	37.44	57.51
Pseudomonadota	5.13	2.27	7.49	7.00
Verrucomicrobia	-	3.20	1.44	2.09
Fusobacteriota	-	-	1.02	-
Actinomycetota	-	-	-	5.37
Others	1.32	1.46	0.76	0.31

Table S4. Relative abundance of gut bacterial communities (≥ 0.01%) at the genus level in healthy and IBD Mexican and Spanish subjects.

Genus	% Relative read abundance MX-H	% Relative read abundance SP-H	% Relative read abundance MX-IBD	% Relative read abundance SP-IBD
Bacteroides	29.00	36.78	27.55	17.58
Prevotella	9.80	-	14.45	-
Alistipes	4.63	6.04	-	3.61
Parabacteroides	2.27	3.97	2.56	-
Agathobacter	2.38	-	-	3.65
Lachnospira	2.91	-	3.38	-
Lachnospiraceae NK4A136	3.28	-	3.66	9.44
Faecalibacterium	4.88	5.74	7.16	7.66
Ruminococcaceae UCG-002	2.50	3.10	-	-
Ruminococcaceae UCG-014	-	2.73	-	-
Ruminococcus	-	4.75	-	-
Phascolarctobacterium	3.26	-	2.50	-
Dialister	2.17	-	2.14	3.84
Barnesiella	-	2.91	-	-
Paraprevotella	-	2.54	-	-
Akkermansia	-	3.09	-	2.09
Sutterella	-	-	3.19	-
Blautia	-	-	-	5.11
Lachnoclostridum	-	-	-	2.14
Escherichia/Shigella	-	-	-	3.24

Bifidobacterium	-	-	-	2.78
Others	32.85	28.28	33.37	38.80

Table S5. Redundancy analysis (RDA) of gut bacterial communities, including eigenvalues, proportion explained, and cumulative proportion by the first 12 RDA axes.

**Component**	**Eigenvalue**	**Proportion explained**	**Cumulative proportion**
RD1	16.6854	0.3195	**0.3195**
RD2	7.6774	0.1470	**0.4665**
RD3	4.88007	0.09344	**0.55995**
RD4	3.8697	0.0741	0.6340
RD5	3.53155	0.06762	0.70167
RD6	2.91323	0.05578	0.75745
RD7	2.65616	0.05086	0.80831
RD8	2.36648	0.04531	0.85363
RD9	2.12712	0.04073	0.89436
RD10	2.10355	0.04028	0.93464
RD11	1.77332	0.03396	0.96859
RD12	1.64020	0.03141	1.00000

Table S6. Loadings of anthropometric and dietary variables on the first two RDA axes explaining variation in gut bacterial communities.

**Variable**	**RDA1**	**RDA2**
BMI	**0.457**	0.119
Simple carbohydrates %	**0.416**	-0.405
Complex carbohydrates %	0.091	**0.425**
Dairy products %	-0.030	**0.481**
Proteins %	-0.141	-0.362
Lipids %	-0.362	-0.150
Fat mass %	**0.451**	0.201
Carbohydrate %	0.363	0.184
Vegetal %	0.073	0.271
Meat %	-0.114	**-0.488**
Lean mass %	-0.244	**-0.524**
Kilocalories intake	**-0.602**	0.334
